# Study on Grouting Repair Effect of Post-Peak Coal Samples

**DOI:** 10.3390/ma19132764

**Published:** 2026-06-30

**Authors:** Yaohui Zhang, Zuqiang Xiong, Xufeng Liu, Chun Wang, Ke Yang, Wanglei Zhang

**Affiliations:** 1 School of Resources and Safety, Henan College of Industry & Information Technology, Jiaozuo 454000, China; zhangyaohui1223@163.com; 2 School of Energy Science and Engineering, Henan Polytechnic University, Jiaozuo 454003, China; 3 State Key Laboratory of Geomechanics and Geotechnical Engineering Safety, Institute of Rock and Soil Mechanics, Chinese Academy of Sciences, Wuhan 430071, China; 4 China Coal Science and Industry Group Taiyuan Research Institute Co., Ltd., Taiyuan 030012, China

**Keywords:** coal, grouting reinforcement, cracking failure, capacity repair

## Abstract

Coal has abundant bedding and joint structures, and most of it exhibits obvious brittle characteristics, which leads to its easy cracking and failure under mining stress. This easily leads to slab cracking and roof collapse in coal mining faces, as well as large deformations in roadways. On-site grouting of fractured coal bodies can effectively prevent these disasters. To reveal this mechanism, this study has first developed a modified ultra-fine cement grouting material and high-pressure continuous grouting system, and then conducted grouting and uniaxial compression tests on post-peak coal samples. Test results indicate that the post-peak residual bearing capacity of grouted coal specimens can recover to 65~85% of the peak strength of intact raw coal. The pre-peak plastic deformation becomes significant, and the post-peak stage exhibits stable strain softening. Grouting is considered to serve to improve the internal stress state of coal samples, act as a ductile grid skeleton, coordinate their internal deformation, and enhance their post-peak bearing capacity.

## 1. Introduction

Coal, as the cornerstone of China’s energy system, occupies an irreplaceable strategic position in ensuring national energy security and underpinning national economic development, with an annual coal output of over 4 billion tons in the past five years [1,2,3]. Safe coal production is not only directly impacting the overall stability of energy supply but also directly concerns the safety of millions of mine workers and the social stability in mining areas, serving as the fundamental prerequisite for the sustainable development of coal mining enterprises. However, the inherent mechanical characteristics of coal pose severe challenges to safe coal mining operations. During the long geological evolution, coal seams have naturally developed many structural planes such as bedding, joints, and fractures due to complex tectonic movements [4,5,6,7]. These inherent defects severely weaken the overall integrity and mechanical properties of the coal mass, causing coal to be highly susceptible to deformation, fracture, and even instability under external loads [8,9,10]. In field mining practice, affected by factors such as mining-induced stress disturbances and complex coal seam occurrence conditions, the coal mass is susceptible to various disasters such as rib spalling and roof collapse [11,12,13]. These accidents not only cause significant equipment damage and production disruptions but also endanger the lives of underground personnel, thereby restricting the safe and efficient development of the coal industry.

To reduce these risks and ensure the stability of coal masses in mining engineering, various reinforcement technologies have been developed and applied, among which grouting reinforcement is a proven effective and widely adopted method. Grouting reinforcement involves injecting specially formulated grout under pressure into the fractures, pores, and structural discontinuities of coal [14,15]. After grouting, the grout infiltrates and fills void spaces, cements fractured coal blocks together, and forms an integrated composite structure, thereby improving the mechanical properties and overall stability of the coal mass. Compared with other reinforcement technologies, grouting exhibits distinct advantages: it can adapt to complex geological conditions, penetrate deep into micro-fractures that are inaccessible to other methods, enable targeted reinforcement, and cause minimal disturbance to the surrounding rock mass [16,17,18]. In field engineering practice, grouting reinforcement has been widely applied in coal mines to address critical engineering challenges including rib support, roof control, and the reinforcement of broken coal pillars [19,20,21,22]. Extensive field applications have confirmed that it is highly effective in reducing mining disasters and improving the safety and efficiency of coal production.

However, despite the widespread application of grouting reinforcement in coal mines, the underlying reinforcement mechanism remains poorly understood—especially for post-peak fractured coal masses that have already lost their structural integrity. This insufficient mechanistic insight severely restricts grouting scheme optimization, parameter design, and accurate effect evaluation in engineering practice. To fill this critical gap, this study takes large-size coal specimens sampled from an actual large-mining-height working face as the research object, and systematically investigates the grouting reinforcement effect and underlying mechanism of these specimens. A modified ultra-fine cement grout was formulated to match the micro-fracture characteristics of concealed adverse geologic bodies in large-mining-height coal faces, and systematic laboratory uniaxial compression tests were performed on grouted specimens. This work aims to reveal the governing laws of grouting on the mechanical response, failure characteristics, and post-peak bearing capacity of fractured coal, providing direct experimental evidence and theoretical support for grouting reinforcement of broken coal masses in field engineering.

## 2. Materials and Methods

### 2.1. Sampling Information

**Figure 1:**
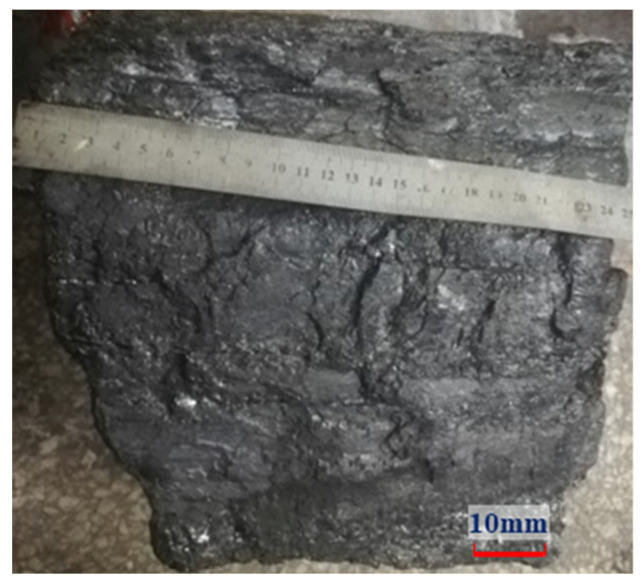
On-site collected No. 3 coal sample.

The coal samples were collected from Zhaozhuang Mine of Jinneng Holding Group, which is located in the Qinshui Coalfield and a key mine of Shanxi Jinmei Group. The mine has a designed production capacity of 8.0 Mt/a and mainly mines the No. 3 coal seam of the Lower Permian Shanxi Formation, which is stably distributed in the mining area. This coal seam is mainly characterized by a layered and linear structure with well-developed endogenous fractures, and is generally classified as a weak coal seam. The bulk density of the coal seam is approximately 1.4 t/m^3^. The specific sampling location is the 1307 large-mining-height working face of the mine. The working face has a strike length of 2084.0 m and a dip length of 233.4 m. The coal seam thickness ranges from 4.60 m to 6.10 m, with an average of 5.36 m. The coal seam in the working face is stable, with no significant thickness variations. The coal seam has a dip angle of 0–5° and a burial depth ranging from 463.9 m to 673.9 m. During the maintenance of the working face, large-sized coal samples were collected from the coal wall (Figure 1). After being packaged, they are transported to the laboratory and cut into four cubic coal samples slightly larger than 150 mm × 150 mm × 150 mm using a CNC saw. Then, they are ground into the required size using a grinding machine. This coal sample is classified as anthracite, characterized by a simple fine-banded texture. It is dominated by vitrain with minor durain, exhibits a submetallic luster, and belongs to the bright coal lithotype.

### 2.2. Testing Program

**Figure 2:**
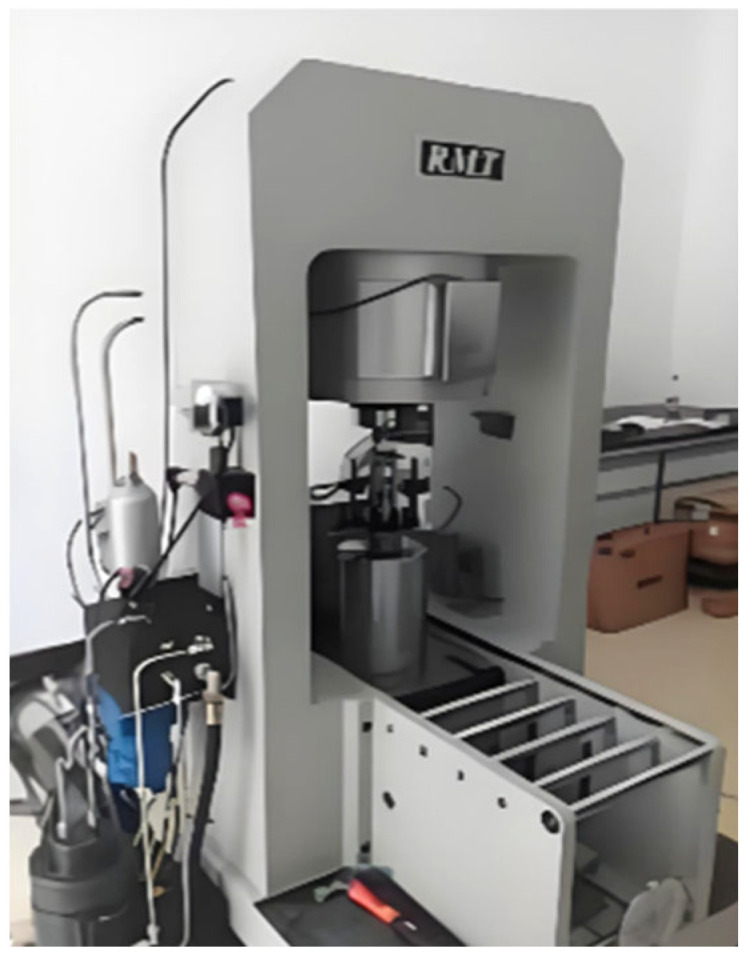
RMT high-stiffness servo-controlled rock mechanics testing machine.

Due to the presence of numerous primary and secondary joints in coal samples, there are significant differences in their mechanical properties. Therefore, this experiment comprehensively evaluates the grouting effect by comparing the mechanical properties of four sets of coal samples before and after grouting. In this experiment, four cube specimens were first tested under uniaxial compression using the RMT high stiffness servo rock mechanics testing machine developed by the Wuhan Institute of Geotechnical Mechanics, Chinese Academy of Sciences (Figure 2). To generate as many fractures as possible in the specimens, the specimens were loaded to a specific post-peak stage, followed by unloading and reloading, with the loading surfaces clearly marked. The tests were performed under displacement control mode, with a displacement rate of 0.001 mm/s. Subsequently, the post-peak specimens were grouted using a self-developed single-liquid high-strength grouting material and a high-pressure grouting system. After that, the grouted coal samples were cured under a standard box for 7 days. Then, the grouted specimens were reprocessed into 150 mm × 150 mm × 150 mm cubic specimens. Finally, aligning with the initial loading surfaces, uniaxial compression tests were conducted again on the grouted coal samples. Through these experimental procedures, the mechanical response of coal samples before and after grouting can be compared, and the grouting repair effect and mechanism can be explored.

### 2.3. Modified Ultra-Fine Cement Grouting Material

**Figure 3:**
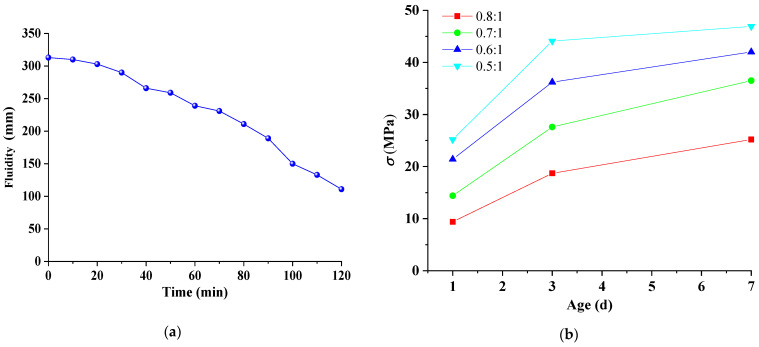
Changes in the flowability and strength of the modified ultra-fine cement grouting materials over time: (**a**) fluidity characteristics at a water cement ratio of 0.6:1; (**b**) uniaxial compressive strength characteristics at different water cement ratios.

**Figure 4:**
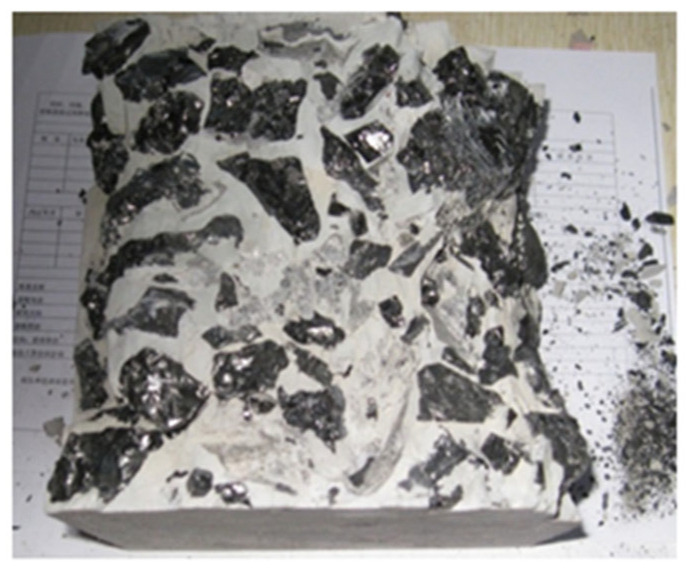
Photographs of coal particles strengthened with grouting materials.

Ordinary Portland cement (OPC) is widely adopted in civil construction engineering, but its inherent defects including long setting time and low early strength restrict its practical application in grouting projects. Moreover, the relatively large particle size of conventional OPC restricts the grout to only penetrate wide fractures. Refining cement fineness can effectively optimize the comprehensive properties of grouting materials, yet it also brings adverse problems such as higher water demand, shortened setting time and grout agglomeration. For this reason, rational admixtures are required to fully leverage the advantages of ultra-fine cement and ensure excellent overall performance of grouting materials. Through extensive laboratory tests, this study formulated a modified ultra-fine cement grouting material for coal mass grouting reinforcement. The developed single-liquid high-strength grouting material is mainly composed of P·O 52.5 Portland cement (Table 1), supplemented with admixtures such as composite retarders, composite early-strength agents, and water reducers. The material is a dry powder, non-toxic and non-corrosive. The recommended water–cement ratio ranges from 0.5:1 to 0.8:1. After being mixed with water, the slurry maintains favorable fluidity within 30–40 min measured by the standard paste fluidity cone mold (Figure 3a). Its initial setting time ranges from 2.5 to 3 h, and the final setting time is approximately 10 h. The strength develops rapidly after solidification, and its key performance characteristics are as follows: good fluidity, enabling it to retain fluidity for an extended period and achieve a favorable diffusion radius; fine particle size, quantitative particle size distribution analysis shows that 99% of the grout particles have a diameter less than 13.13 μm by volume, corresponding to a D_99_ value of 13.13 μm, allowing it to be injected into fine fractures; high and rapid strength development, for instance, at a water–cement ratio of 0.6:1, the 1-day compressive strength is 21.5 MPa, the 3-day compressive strength is 36.4 MPa, the 7-day compressive strength is 42.3 MPa, and the final strength can reach 45 MPa (Figure 3b). The grouting material was used to reinforce coal blocks, as illustrated in Figure 4.

### 2.4. Grouting System and Grouting Process

**Figure 5:**
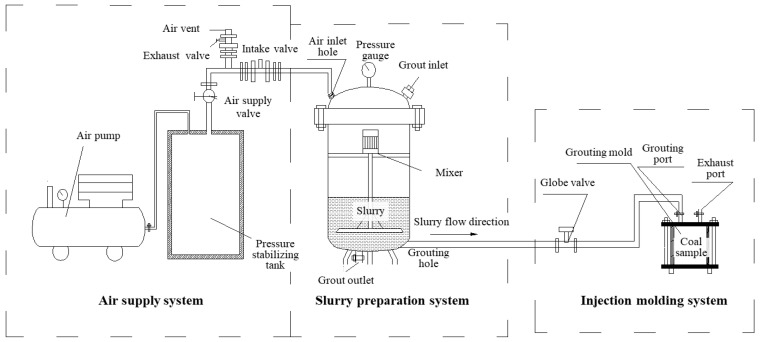
Schematic diagram of the grouting system.

To facilitate the smooth execution of laboratory grouting tests, a custom-designed continuous high-pressure grouting device was developed. It comprises three components: an air supply system, a slurry preparation system, and an injection molding system, as shown in Figure 5. The air supply system includes an air pump and a pressure stabilizer tank. The slurry preparation system consists of a high-pressure stirring tank. The injection molding system comprises a mixer, a flow meter, and a high-pressure grouting mold. This device enables stable and continuous high-pressure grouting. The device has the following key advantages: ① High pressure resistance (up to 8 MPa), ensuring effective grouting outcomes; ② Stable pressure output, achieved by using an air pump for air supply and a pressure stabilizer tank to regulate pressure and store air, ensuring continuous, stable pressure supply; ③ Sealed stirring in the slurry preparation system: a sealed stirring device operates continuously during grouting to maintain consistent performance.

**Figure 6:**
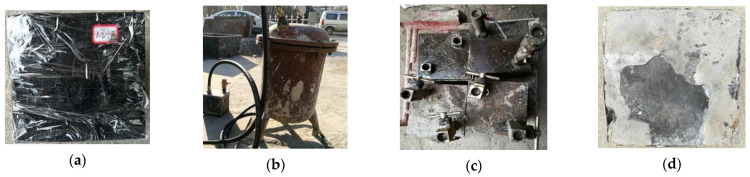
Photo of the grouting process: (**a**) post peak coal sample; (**b**) high-pressure stirring tank; (**c**) grouting mold; (**d**) the grouted solid after demolding.

Prior to grouting, post-peak fractured coal specimens are secured inside the customized grouting mould. Well-blended grout is fed into the feeding system under sustained agitation. Operators adjust the air pump and assorted valves to build up pressure, keeping the stabilizer tank stabilized at 5 MPa for a 2 min holding period. Subsequently, the globe valve and mould exhaust port are opened to launch formal grouting. The exhaust opening is shut once steady, uniform slurry flows out, and grouting is terminated when the flowmeter records zero flow rate. After injection, relevant air and cut-off valves are closed for system depressurization, and the pressure tank together with connected pipelines is rinsed with clean water. The assembled grouting apparatus is presented in Figure 6.

**Figure 7:**
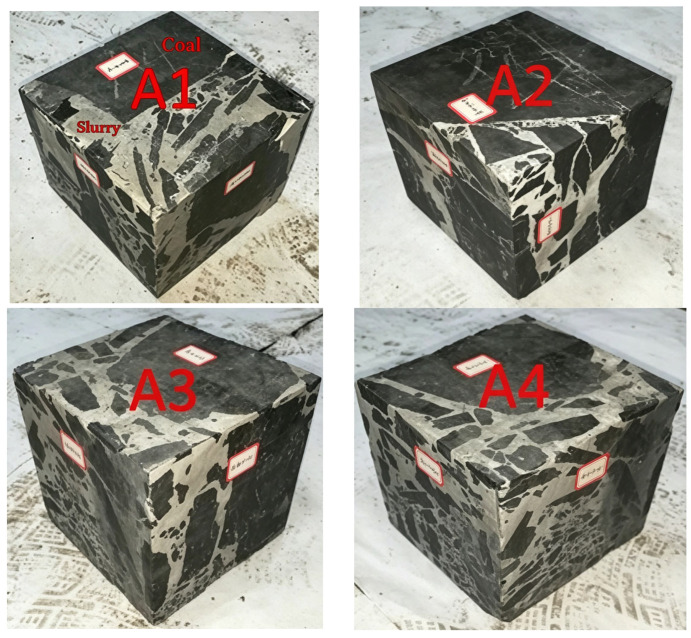
Photo of the samples after grouting.

The grouted coal samples were demolded one day after grouting and subsequent solidification. Following demolding, the grouting-reinforced coal samples were cured under standard indoor curing conditions for seven days. They were then re-machined into standard 150 mm × 150 mm × 150 mm cubic specimens, as illustrated in Figure 7. The grouted coal samples exhibited good integrity; visually inspected, the fractures in the post-peak coal samples are fully filled with the grout. This demonstrated that the aforementioned grouting scheme can fully meet the grouting technical requirements.

## 3. Results

### 3.1. Wave Velocity Characteristics

Longitudinal wave velocity provides a quantitative indicator of the degree of crack development in a rock specimen. To investigate the internal crack development and grouting reinforcement effect of coal samples under three conditions: pre-loading, post-peak loading, and post-grouting, longitudinal wave velocity tests were conducted on the coal samples in three directions for each condition, with the results presented in Table 2. The test results show significant dispersion in longitudinal wave velocities among the three directions of coal samples before loading. For sample A3, the wave velocity of the 1-1 direction is only 1449 m/s, while that of the 2-2 direction reaches 2220 m/s. This is primarily because coal is a typical layered heterogeneous material with substantial differences in mechanical properties across different directions, which is evident in the significantly higher longitudinal wave velocity in the direction parallel to the bedding compared to that in the direction perpendicular to the bedding. After loading, the longitudinal wave velocity of the coal samples decreases considerably. For sample A1, the average longitudinal wave velocity before loading is 1956 m/s, which drops to 1318 m/s after loading, representing a reduction of 44%, and similar patterns are observed in other coal samples. Following grouting reinforcement, the average longitudinal wave velocities increase significantly. Among them, the longitudinal wave velocities of samples A3 and A4 even exceed those of the original coal samples, while those of A1 and A2 are roughly equivalent to their pre-loading longitudinal wave velocities. The test results demonstrate that the inherent heterogeneity of coal samples causes variations in longitudinal wave velocities across different directions; extensive crack propagation induced by post-peak loading leads to a substantial decrease in longitudinal wave velocity; and grouting reinforcement effectively fills the internal cracks of post-peak loaded coal samples, enhances their integrity, and thereby significantly increases their longitudinal wave velocities.

### 3.2. Stress–Strain Curve

**Figure 8:**
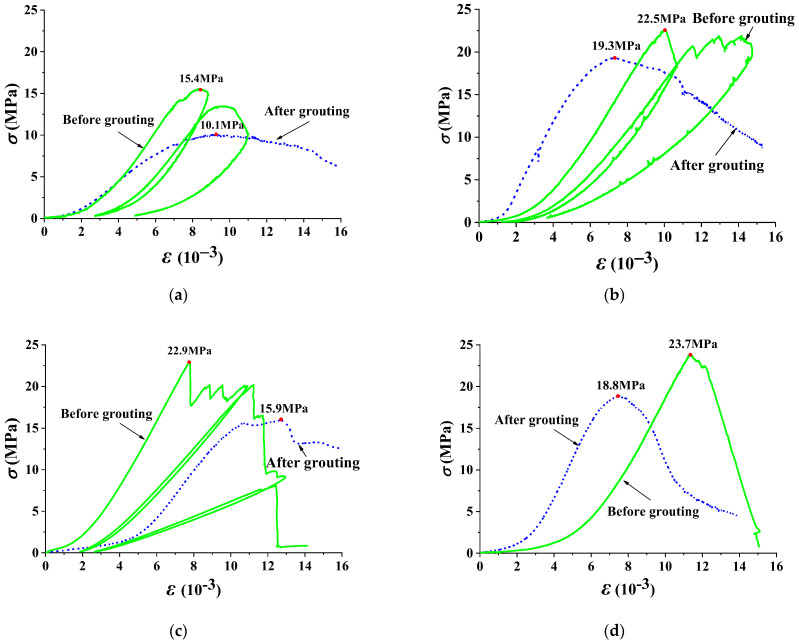
Stress–strain curves of uniaxial compression for coal samples before and after grouting: (**a**) A1; (**b**) A2; (**c**) A3; (**d**) A4.

Figure 8 presents the full stress–strain curves of uniaxial compression for coal samples before and after grouting. Sample A1 exhibited an obvious pre-peak plastic deformation stage. After unloading at the post-peak stage immediately following the peak stress, the reloading stress peak was slightly lower than that before unloading. The sample exhibited similar plastic softening properties, matching the curve shape induced by strain softening. After grouting subsequent to unloading, the sample displayed more significant pre-peak plastic deformation during reloading. The peak stress region was flatter, showing approximately ideal plastic properties. These deformation characteristics were markedly different from those of natural coal samples. Compared with Sample A1, Samples A2 and A3 exhibited significant brittle properties. They displayed no obvious pre-peak plastic deformation stage, and multi-stage stress drops occurred after the peak. After grouting subsequent to unloading, their deformation characteristics during reloading were similar to those of Sample A1, showing ductile failure properties. Even with large post-peak deformation, they still retained significant residual bearing capacity. Unlike Samples A1, A2, and A3, Sample A4 underwent brittle unstable failure without undergoing loading-unloading cycles. Due to insufficient fracture development, its post-peak stress–strain behavior after grouting was similar to that of ordinary soft rock, displaying stable strain softening characteristics. Overall, intact coal samples exhibited little pre-peak plastic deformation, and they displayed multiple post-peak stress drops, showing brittle failure properties. After grouting, pronounced pre-peak plastic deformation was observed, and stable post-peak strain softening occurred, indicative of progressive ductile failure properties.

### 3.3. Bearing Capacity Characteristics

By comparing the stress–strain curves of grouted coal samples and original coal samples, the peak stress of the grouted coal samples generally did not exceed that of the original coal samples (Figure 8). The peak stresses of the grouted coal samples for A1, A2, A3, and A4 were 65%, 85%, 69%, and 79% of those of their corresponding intact counterparts, respectively. Samples A1 and A2 were unloaded at the post-peak stage immediately adjacent to the peak stress, and their peak stress after grouting and reloading was equivalent to that before unloading. Sample A3 was grouted after it reached the residual strength stage, and the sample’s peak stress after grouting was much higher than its residual strength before grouting. Sample A4 underwent brittle unstable failure with slight post-peak deformation, and its bearing capacity was also significantly restored after grouting. Grouting also exerted a notable influence on the post-peak bearing capacity of the samples. Original coal samples usually exhibited an obvious stress peak, with multi-stage stress drops after the peak, showing significant brittle failure characteristics. After grouting, the peak stress of the samples was usually less pronounced, and the post-peak stress decreased slowly with increasing deformation. The samples still retained considerable residual bearing capacity even after undergoing substantial post-peak deformation.

### 3.4. Failure Characteristics

**Figure 9:**
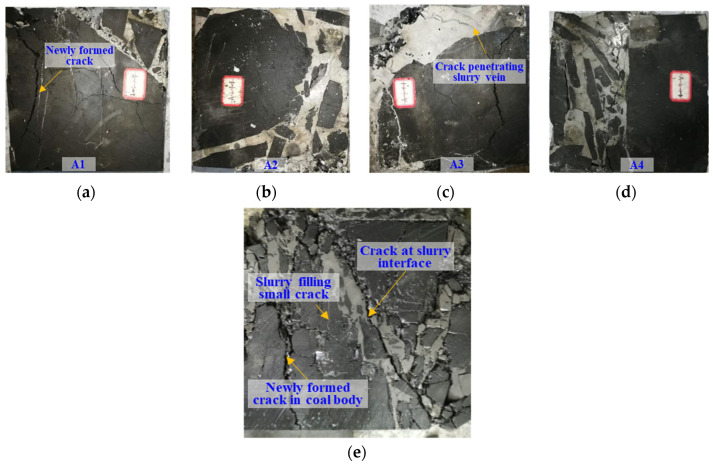
Failure mode of grouted solid: (**a**) A1; (**b**) A2; (**c**) A3; (**d**) A4; (**e**) cross-section of Sample A1.

The failure modes of grouted post-peak coal specimens are illustrated in Figure 9. Under loading, the grouted coal samples exhibited predominantly compression-induced tensile failure. Tensile cracks were more common in the intact coal matrix, with some failures occurring at the contact interface between coal and grout; overall, the hardened grout stone itself sustained relatively minor damage. This was mainly attributed to the elastic mismatch between the grout stone and the coal sample. The elastic modulus of the coal matrix was higher than that of the grout stone. During loading, the coal matrix underwent minimal deformation, acted as the main load-bearing component, and failed first. In contrast, the grout stone mainly underwent flexible yielding under pressure, and the elastic mismatch between the two also made the interface a weak zone prone to crack initiation.

Sample A1 was cut longitudinally, as shown in Figure 9e. After grouting, the grout was able to infiltrate and fill most internal fractures of the post-peak loaded coal sample and even penetrate into fractures smaller than 1 mm, indicating that the grouting material is capable of effectively reinforcing broken coal masses. It was also found that fractures occurred both inside the coal sample and at the grout–coal interface, suggesting that the bearing capacity of the reinforced composite depends on the bearing capacity of the original coal and the bond strength of the grout–coal interface.

## 4. Discussion

Based on the experimental results, grouting reinforcement significantly enhanced the post-peak bearing capacity of post-peak fractured coal samples, but could not restore their peak bearing capacity to the level of intact coal samples, which is similar to the research results of [23,24]. This finding differs from the common empirical perception among field engineers that grouting can raise the strength of broken coal beyond the original state. The reason is that the coal blocks remain the primary load-bearing structure, and the grout only optimizes the stress condition of the broken coal mass rather than replacing its load-bearing role. Despite failing to recover the peak strength of intact coal, grouting provides a critical improvement to the mechanical behavior of post-peak coal, which is directly quantifiable and verifiable through comparative analysis of pre- and post-grouting stress–strain curves.

**Figure 10:**
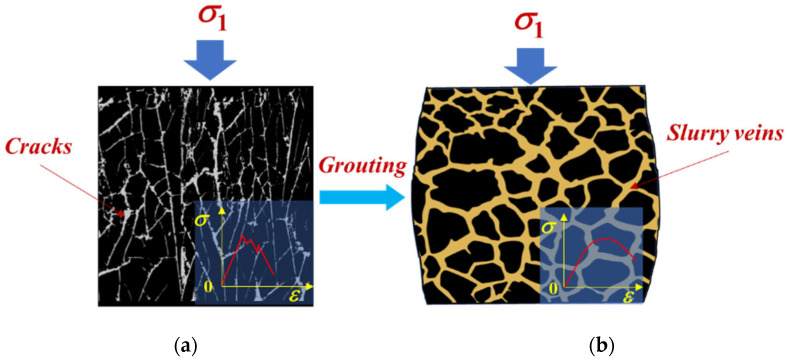
Schematic interpretation of the proposed grouting reinforcement mechanism for post-peak fractured coal: (**a**) brittle failure behavior of ungrouted specimens; (**b**) proposed resilient mesh-like structure coordinating internal deformation after grouting.

The ungrouted post-peak coal samples exhibited remarkable brittleness governed by the inherent properties of coal, and after brittle fracture of the coal sample occurs, the coordinated deformation conditions that mutually constrain both sides of the fractured surface are disrupted [25,26]. Multiple micro-uniaxial compression units separated by fractures emerged inside the post-peak sample, and cracking will further develop and evolve with loading, which is reflected in the random fluctuation trend of the stress–strain curve. In contrast, after grouting, the slurry fully filled the internal fractures and optimized the stress distribution. The hardened grout has a relatively low deformation modulus compared to coal matrix, which enables it to coordinate with the deformation of adjacent coal blocks, mitigate local stress concentrations, and appears to form a resilient mesh-like structure within the fractured coal mass (Figure 10). The quantitative characteristics of the post-grouting stress–strain curves align well with this proposed interpretive framework: grouted specimens exhibited pronounced pre-peak plastic deformation, a broadened and flattened peak region, and gradual post-peak strain softening behavior, in stark contrast to the catastrophic brittle failure observed in ungrouted coal samples. Collectively, these observations suggest that grouting modifies the internal stress state of post-peak damaged coal. The grout-filled fractures appear to function as a ductile mesh-like structure that coordinates the overall deformation of the coal mass, transforming the failure mode from abrupt brittle disintegration to a more uniform and progressive ductile failure process. This effect likely contributes to the improved post-peak bearing capacity and enhanced stability of fractured coal masses observed in our experiments.

From the failure modes of the samples, it can be observed that many cracks develop along the interface between the consolidated grouting material and the coal mass, indicating that the grout–coal interface remains a weak zone [27]. This is mainly because coal contains a large amount of organic matter, which endows the coal surface with strong hydrophobicity. As a result, fewer hydration products of the grouting material are formed at the grout–coal interface, ultimately leading to a loose structure and weak cementation in the grout–coal interface zone. Studies have shown that adding interface wetting agents can significantly improve the structure of the grout–coal interface, thereby enhancing the strength of the consolidated grouting material. This also represents our future research direction.

Despite the coal specimens being collected from the same coal seam and working face with relatively consistent internal structure, and the adoption of 150 mm cubic large-size specimens to effectively reduce heterogeneity-related discreteness, only four groups of specimens were tested in this study due to the extremely high difficulty and prohibitive cost of acquiring large intact bulk coal samples from underground working faces. In addition, another important limitation lies in the interpretive nature of the proposed resilient mesh-like reinforcement mechanism. As conventional macroscopic mechanical tests cannot directly visualize the three-dimensional spatial distribution and morphological characteristics of the hardened grout skeleton within the fractured coal mass, this mechanism is primarily inferred from the comprehensive analysis of indirect experimental evidence, including wave velocity recovery, full-process stress–strain behavior, post-peak residual strength retention, and macroscopic failure surface characteristics. Therefore, the conclusions drawn in this study are representative of the studied mine site but have limited generalizability under different geological conditions and grouting material systems. In subsequent work, we will conduct more systematic tests with an increased number of specimens covering various coal masses and geological conditions to enhance the generalizability of the findings. Meanwhile, advanced internal structure observation techniques will be employed to directly observe the internal grout network structure and quantitatively characterize its geometric parameters, which will provide definitive experimental evidence to validate and further refine the proposed reinforcement mechanism.

## 5. Conclusions

This paper conducted a systematic study on the grouting reinforcement effect and mechanism of broken coal. First, a modified ultra-fine cement grouting material and corresponding grouting equipment were developed, and then the mechanical responses of coal samples before and after grouting were investigated through indoor uniaxial compression tests. The main conclusions are as follows:

1. External loading generates internal fractures and markedly reduces coal P-wave velocity. Grouting fills inner cracks, restores specimen integrity and substantially raises longitudinal wave velocity, with a maximum increase rate of 1.44 times.
2. Unreinforced coal specimens show negligible pre-peak plastic deformation, sharp stress peak and abrupt multi-stage post-peak stress decline, presenting typical brittle failure. After grouting treatment, samples gain obvious pre-peak plastic deformation, a gentler peak stress and stable post-peak strain softening; they maintain high residual bearing capacity even under large post-peak deformation; the post-peak residual bearing capacity can recover to 65% ~ 85% of the peak strength of intact raw coal.
3. Grouted coal specimens predominantly suffer compression-induced tensile fracturing. Cracks mostly develop within coal matrix or along coal–grout bonding interfaces, whereas the grouting solid remains largely intact. Accordingly, the overall bearing capacity of reinforced specimens is controlled by raw coal strength and interfacial adhesion between coal and grout.
4. Grouting effectively enhances the post-peak bearing capacity of fractured coal but cannot restore the peak strength of intact raw coal. It optimizes the internal stress field inside specimens and forms a ductile skeleton to coordinate heterogeneous deformation, thus improving residual load-bearing performance after peak stress.
5. The modified ultra-fine cement grouting material possesses favorable infiltration capacity to penetrate microscale fractures inside coal. Nevertheless, the bonding interface between grout and coal still constitutes the primary weak plane of the reinforced body. Accordingly, adopting suitable interface modifying additives to upgrade interfacial mechanical performance will be a vital orientation for subsequent research.

## Figures and Tables

**Table 1:** Chemical and Mineral Composition of PO 52.5 Cement, %.

SiO_2_	Al_2_O_3_	Fe_2_O_3_	CaO	MgO	f-CaO	K_2_O	SO_3_
21.29	6.03	3.29	65.37	1.09	0.50	0.76	1.67

**Table 2:** Changes in wave velocity of coal samples before and after loading and grouting.

No.	Original Coal Sample				Post-Loading Coal Sample				Post-Grouting Coal Sample			
	1-1	2-2	3-3	Average (m/s)	1-1	2-2	3-3	Average (m/s)	1-1	2-2	3-3	Average (m/s)
A1	1932	1929	2008	1956	1349	1250	1356	1318	1713	2073	1931	1905
A2	1637	1951	1897	1828	1420	1382	1372	1391	1729	1857	1917	1834
A3	1449	2220	1830	1833	1210	1460	1342	1337	1920	2071	2115	2035
A4	1927	1914	1305	1715	1332	1198	1120	1216	2003	2144	1761	1969

## Data Availability

All data used during the study appear in the submitted article.
